# The physics of unwound and wound strings on the electric guitar applied to the pitch intervals produced by tremolo/vibrato arm systems

**DOI:** 10.1371/journal.pone.0184803

**Published:** 2017-09-21

**Authors:** Jonathan A. Kemp

**Affiliations:** 1 Music Centre, University of St Andrews, St Andrews, Fife, United Kingdom; 2 SUPA, School of Physics & Astronomy, University of St Andrews, St Andrews, Fife, United Kingdom; Goethe-Universitat Frankfurt am Main, GERMANY

## Abstract

The physics of wound and unwound strings on the electric guitar are presented here, and the pitch intervals produced by the movements of a Fender Stratocaster tremolo unit are explained. Predicted changes in pitch sensitivity of different strings are given, and experimentally verified, for changes in saddle height, the distance of string free to move behind the nut and ratio of diameters/masses of the core and windings of wound strings. Also, it is shown that changes to the gauge of strings (assuming the string tension is sufficient for linear behaviour and in absence of changes to other construction details) don’t alter the pitch intervals produced by a given angle of tremolo arm use assuming the instrument is set up with the same sounding pitches and starting bridge angle. It is demonstrated that it not possible to equalise the relative sensitivity of unwound steel stings on a Fender Stratocaster type tremolo unit through string construction techniques. The ratio of core to winding mass in the string, on the other hand, was found to be a very powerful design parameter for choosing the sensitivity of the string to tremolo arm use and standard pitch bends. For instance, the pitch intervals produced by operation of tremolo arm for wound strings may be made to approximately match that for one of the unwound strings if they share very similar core gauges (assuming the winding masses are chosen to give approximately the same tension at their sounding pitches). Such a design, only available currently by custom order, also delivers the optimum equalisation in sensitivity of strings for standard string bends (due to these also being produced by altering the length of the string to generate changes in tension and therefore pitch).

## Introduction

The physics of string bending techniques have been covered recently by Grimes [1]. When fretting a note on the guitar, the string is pressed directly towards the fretboard such that it contacts a fret with enough force to ensure the string is, to the first approximation, fixed both at the fret and at the bridge (but without pressing so hard as to increase the tension in the string significantly by over-stretching it). Sounding the note then involves plucking the string between these two points. Vibrato effects (player controlled oscillations in pitch) on a guitar can be created by a number of means, including forcing the string tension to adjust by dragging the string either along its length or transverse to the string. Dragging the string in the longitudinal direction is capable of producing both raising and lowering of pitch with respect to that sounded without dragging and is the preferred method for most classical guitarists. On the other hand dragging the strong in the direction transverse to its length and parallel to the fret (allowing for modulation to pitches above the original) is the conventional string bending technique for many guitarists playing in popular music styles including electric guitarists. A combination of these techniques may also be employed.

Another significant method of creating vibrato effects on the guitar involves the use of what has become variously known as a “tremolo arm”, “vibrato arm” or “whammy bar”. The player applies force to the tremolo arm in order to move the bridge and adjust the tension of the strings and thus the sounding pitches. Since modulating the force on the arm produces vibrato (modulations in frequency) the term “vibrato arm” is technically appropriate and the first commercially successful device was named the Bigsby Vibrato Tailpiece (patent filed by Paul Bigsby in 1952) [2]. In this design a spring-mounted tailpiece (on which the string ends were mounted and with an arm attached) allows the player to modulate the tension of the strings that passed over a fixed bridge.

Since the term “tremolo” is usually reserved for modulations in amplitude (and this is something that is often achieved using electronic effect units), the term “tremolo arm” is a misnomer for mechanical pitch adjustment systems, but this nonetheless became standard terminology through Leo Fender’s introduction of the Fender Stratocaster and its “synchronized tremolo” in 1954 [3]. This design effectively features the tailpiece (named the tremolo block) and bridge screwed together (and thus synchronized) while still being spring loaded. This action allowed for improved tuning stability over a wider range of pitch adjustment in relation to earlier designs. Adjusting the tension (whether by tremolo arm use or conventional string bending) necessarily means that the string will move over the nut, string trees, and possibly at points of contact with the bridge. The string may therefore come to rest with slightly imbalanced tensions on either side of any point of non-negligible friction, resulting in tuning problems.

This particular source of tuning problems was largely solved with the invention of the Floyd Rose Locking Tremolo (patent filed in 1977 [4]) in which the string is rigidly clamped to the nut and to the saddles in the bridge. While the Floyd Rose design enjoys enduring popularity, the Fender design is still more popular today overall, and this may be simply due to its unobtrusive “vintage” look and relative simplicity (while locking nut designs usually require unlocking using Allen keys before restringing for instance).

The pitch of different strings deviate by different relative intervals during tremolo arm operation in the designs of Fender and Floyd Rose, meaning that in-tune chords become discordant when significant tremolo arm usage is applied. Ralph Jones developed a moveable tailpiece with strings mounted at adjustable distances with respect to the pivot in order to achieve chords that could be adjusted in tune (patent filed in 1965 [5]). Marketed under the name “Calibrato” and fitted to guitars by his short-lived Micro-frets company, this system was unique in achieving musical “pedal steel” style multiple-string bends on the guitar without needing specially calibrated strings. Ned Steinberger invented a system, in a patent filed in 1985, for a calibrated string angles and lengths at the bridge to deliver equal relative pitch intervals for a given angle of tremolo arm movement [6]. Marketed (under the name TransTrem) in a format that allowed for very accurate pitch transposition in semitone intervals in addition to in-tune tremolo arm bends, this system was employed by a number of players in spite of the complexity of setting up the system and doubts about long term availability of the replacement parts and calibrated double ball-end string sets. Later designs for custom hardware to control relative pitch intervals under tremolo arm use have included the Washburn Wonderbar (which appears to have made use of a patent [7]) and the ChordBender (also subject of a patent [8]).

The properties of tremolo arm systems, how they should be set up, the relative intervals with tremolo arm use and problems and solutions for tuning stability are popular topics of discussion with electric guitarists today. Tremolo arm use is dealt with fleeting by Grimes [1] without going into detail on issues such as the relative tuning of different strings for a given tremolo arm motion. This article also ignored the effect of strings slipping across the nut on bending strings and this is likely to have led to the strain in the string being over-estimated given that the experiments were not performed on an instrument with a locking nut. A change in geometric length for the string leads to the string slipping across the nut to approximately equalise the tension, meaning that the strain will be around 17% lower when the distance of string available between the nut and the tuning peg is around 17% of the sounding length. Accordingly, the results gave experimentally determined values for the Young’s modulus of the unwound strings (171—189 GPa) are lower than the expected values for steel (200—220GPa [9]). The experimentally derived values of Young’s modulus for the wound or composite strings were also measured as being even more markedly lower and decreasing with frequency due to not relating the results to the properties of the core and winding.

While the topic has been explored by various patents, to the author’s knowledge, the only treatment of the physics of tremolo arm usage that accounts for relative tuning of different strings on tremolo arm usage is to be found in a student project by Jeremy Ozer [10] at Carnegie Mellon University. This discussion gives a good first approximation formula for the frequency against tremolo arm angle (except for omitting the straightforward formula relating the “spring constant” of the string to the Young’s modulus, cross-sectional area and sounding length). It is stated that no string movement at the nut is being assumed so that the analysis is valid for a locking nut. An appropriate graph of playing frequency (on a linear frequency scale) against tremolo arm angle is also given predicting the large variation in pitch changes across different strings. On the other hand, “ideal” graph of parallel lines on a linear frequency scale is also given suggesting that equal pitch intervals would be achieved on different strings if the frequencies of the strings change by equal number of Hz. This is incorrect as equal ratios of frequency would be ideal in this regard.

It is worth examining the physics of the tremolo arm systems in order to provide information for players, technicians, instrument manufacturers, string manufacturers in addition to musical acousticians and those interested in accurate physical modelling synthesis of this type of system. Naturally this also involves a clear treatment of how the behaviour of wound strings relates to the fundamental properties of the material construction and the relative masses in the core and windings. The subject of how string slippage at the nut alters the relative pitch intervals is also an important part of this study.

## Tremolo system operation

**Fig 1:**
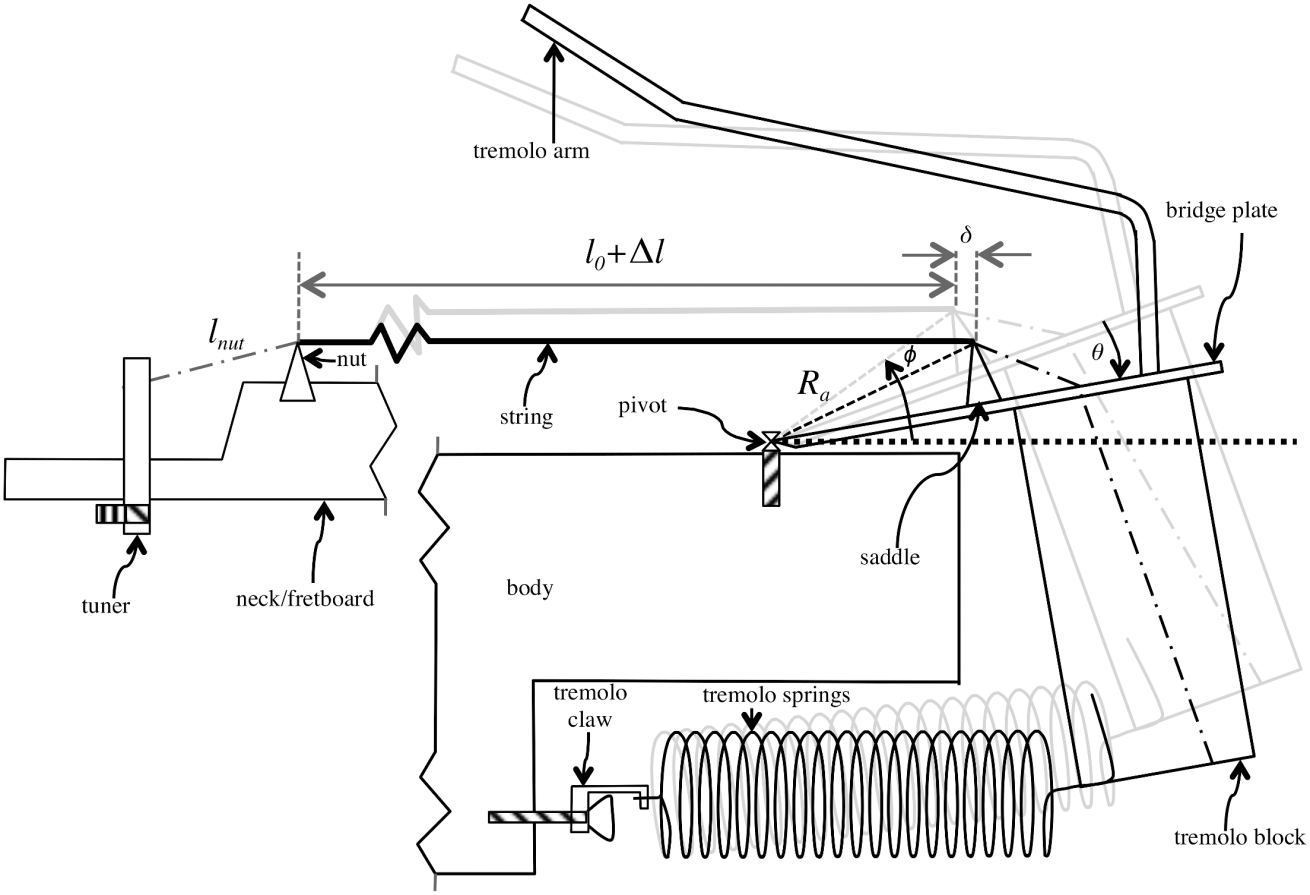
Cross-section of an electric guitar showing the operation of a tremolo system (not to scale). The vibrating length (*l*_0_ + Δ*l*) of the tuned up string is shown as a thick grey line. When the tremolo arm is raised (away from the body), the bridge plate, saddle and tremolo block are all rotated by an angle of *θ* around the pivot. The resulting sounding length, shown as a thick black line, is increased by a distance *δ*.

A typical tremolo system in an electric guitar, based on the design of Leo Fender [3], is shown in Fig 1. For clarity, the size of the saddle and the distance between the saddle and pivot have been over-emphasised in relation to the other dimensions in the figure. Moving the tremolo arm results in an equal angle of rotation, *θ*, around the pivot for the saddles, bridge plate and tremolo block. The initial position of the moving components are shown in grey while their position after applying a force to move the tremolo arm tip away from the body are shown in black. After tuning and before the player applies force to the tremolo arm, the sounding length of the string is *l*_0_ + Δ*l*. This portion of string is defined as having had a length of *l*_0_ before being tuned up to pitch using the tuner (but *l*_0_ is not equal to the sounding length because the string stretches and slips across the nut and saddles and may rotate the position of the bridge when the tuner is used to tune up the string to the desired pitch). After using the tremolo arm, the sounding length of the string is further increased by a distance *δ*, further increasing the tension and therefore sounding pitch. The sections of string not contributing to the sounding length (to the left of the nut and to the right of the saddle) are denoted by dash-dot lines. Note that pressing the tremolo arm tip towards the body produces a rotation in the opposite direction (giving a negative value for *θ*) resulting in a decrease in pitch. In a double locking Floyd Rose system, the string is fixed at the nut and saddle meaning that the geometric value of *δ* corresponds exactly to the change in length of the portion of string responsible for the sounding length (and this follows from the position of the point of contact with the saddle with respect to the pivot before, (*R*_*a*_, *ϕ*), and after, (*R*_*a*_, *ϕ* − *θ*), the change using polar coordinates). In the older Fender design the string slips over the nut and saddles to (within the limits of friction) equalise tension meaning that the lengths of string either side of the nut and saddle must be taken into account.

While it is sometimes stated that “string tension on the top equals spring tension on the underside of the guitar” [11], this is not a technically correct use of the word “tension” since the tensions in question are at different distances from the axis of rotation. Correcting this statement involves simply replacing the word “tension” with the word “torque” or “moment” [10]. The tremolo bridge acts analogously to a mechanical balance and unequal forces may balance the bridge if they are different distances from the pivot such that the torque (defined as the distance of the force from the pivot multiplied by the force) is the same on each side. Standard tremolo designs today have a larger total string tension than total spring tension because the strings are applying the force closer to the pivot than the springs.

The design of Leo Fender involves six strings mounted under similar tension and this tension applies a torque (twisting force) around the pivot (which is defined as the line connecting the points of contact between knife edges in the bridge plate and the screws mounted in the body). The support posts for vintage Fender Stratocaster designs feature six screws while the current American Standard Fender Stratocaster features two (in order to give fewer points of friction). Assuming that the tremolo arm has no additional force applied to it and that the instrument has been set up for floating operation (meaning that the only point of contact between the bridge plate/tremolo block and the body is at the mounting posts) then the torque generated by the springs should be equal and opposite to the torque generated by the strings. There are usually between two and five springs mounted in such a tremolo system in order to balance the the torques.

Adjustment of the rest position of the bridge plate may be achieved by tightening or loosening the screws attaching the tremolo claw to the body. If the screws are tightened until the spring torque overcomes the string torque sufficiently for the bridge plate to rest against the body then it is only possible for the player to adjust the playing pitch downwards (only negative values are possible for *θ* and *δ*). Setting the instrument for floating bridge operation (as pictured in the original Fender patent [3]) involves the bridge plate sitting in a position where both positive and negative pitch deviations are possible (therefore *θ* and *δ* may be positive or negative).

## Sounding frequency and strain on unwound strings

In order to explain the behaviour of tremolo systems in more detail it is worth first reviewing the relationship between string elongation and tensions. The tension in a plain or unwound string is given by the equation:

$$\begin{array}{c} T_{f}=EA_{0}\frac{\Delta l}{l_{0}} \end{array}$$ (1)

where *A*_0_ is the cross-sectional area of the unstretched string, *l*_0_ is the rest length of the portion of the string that has a sounding length of *l*_*f*_ = *l*_0_ + Δ*l* after being tuned up to normal playing tension, *T*_*f*_, and *E* is the Young’s modulus. Note that this assumes that the Young’s modulus measured for nominal stress (constant cross-section) is valid. In reality the linear regime may be exceeded to some extent and this may occur due to the string cross-section contracting in diameter as the string is lengthened (due to the Poisson effect) and due to nonlinearity in the stress-strain graph for the particular material used.

The fundamental frequency of a stretched string of sounding length *l*_*f*_ = *l*_0_ + Δ*l* as a function of the tension is [1]:

$$\begin{array}{c} \nu_{f}=\frac{1}{2l_{f}}\sqrt{\frac{T_{f}}{\mu }} \end{array}$$ (2)

where *μ* is the mass per unit length of the string.

The mass per unit length does not remain precisely constant when the string is stretched [1]. Defining the mass per unit length of the unstretched string as *μ*_0_, the mass per unit length after stretching will be:

$$\begin{array}{c} \mu =\mu_{0}\frac{l_{0}}{l_{f}}=\rho_{0}A_{0}\frac{l_{0}}{l_{f}}. \end{array}$$ (3)

where *ρ*_0_ is the density and *A*_0_ is the original cross-sectional area of the string when unstretched. The sounding frequency is then given by

$$\begin{array}{c} \nu_{f}=\frac{1}{2l_{f}}\sqrt{\frac{E\frac{\Delta l}{l_{0}}}{\rho_{0}\frac{l_{0}}{l_{f}}}}, \end{array}$$ (4)

or

$$\begin{array}{c} \nu_{f}=\frac{1}{2l_{f}}\sqrt{\frac{E(\frac{\Delta l}{l_{f}})}{\rho_{0}{\left(1-\frac{\Delta l}{l_{f}}\right)}^{2}}}. \end{array}$$ (5)

Squaring both sides and solving the resulting quadratic equation gives a solution for the engineering strain on the string as:

$$\begin{array}{c} \frac{\Delta l}{l_{f}}=1+\frac{E}{2\rho_{0}{\left(2l_{f}\nu_{f}\right)}^{2}}\left(1-\sqrt{1+\frac{4\rho_{0}{\left(2l_{f}\nu_{f}\right)}^{2}}{E}}\right). \end{array}$$ (6)

The binomial expansion of this for Δ*l* ≪ *l*_*f*_ gives:

$$\begin{array}{c} \frac{\Delta l}{l_{f}}=\left(\frac{\rho_{0}{\left(2l_{f}\nu_{f}\right)}^{2}}{E}\right)-2{\left(\frac{\rho_{0}{\left(2l_{f}\nu_{f}\right)}^{2}}{E}\right)}^{2}+5{\left(\frac{\rho_{0}{\left(2l_{f}\nu_{f}\right)}^{2}}{E}\right)}^{3}-\ldots \end{array}$$ (7)

Note Eqs (6) and (7) imply that the fractional length change required to bring up an unwound string (of given Young’s modulus and volumetric density) to a given frequency is independent of the diameter or cross-sectional area of the string (though the tension required to achieve this strain would be proportional to the diameter squared if it was circular in cross-section). The strain, and therefore the length of string taken onto the tuning peg, will increase by a factor of around four, to the first approximation, for an unwound string tuned an octave higher if there was no slack in the string prior to tightening to bring it up to pitch. This effect is most noticeable through the larger distance of winding on the thinnest string (a high E in standard tuning) when tuning up from a position of no slack using tuning pegs that clamp onto the string (known as locking tuners), and in the increased rate of breakage of in strings designed to play higher pitches.

## Theory for wound strings

The D, A and low E (lower pitch) strings on the electric guitar are usually wound. We will consider the most common case, known as round wound strings, which consist of a core (which may have a circular or hexagonal cross-section) wrapped with cylindrical wire. This is done in order that the degree of stretching involved in getting them up to pitch is large enough to give the desired approximately linear behaviour (where the pitch is close to being largely independent of the amplitude of vibration) and to prevent excessive resistance to flexing (which would lead to inharmonicity) [12]. The G string was also usually wound in the 1950s (when the Fender Stratocaster tremolo was first invented) but most players have used unwound G strings since lower tension string sets become popular from the 1960s onwards. The winding acts to increase the mass per unit length (hence allowing for lower resonance frequencies).

The tension in wound strings is, to the first approximation, produced by the stretching of the core, as the (helical or spring-shaped) winding is angled and doesn’t stretch along its length as significantly. It worth proving the limited extent to which the winding contributes to the overall axial tension of the string.

### Tension within windings

Assuming the winding behaves like a helical spring consisting of *N*_*a*_ active coils of wire diameter *d*_*w*_, with a mean diameter of *D*_*w*_ with respect to the axis of the core, and modulus of rigidity *G*, the spring constant will be [13]:

$$\begin{array}{c} k=\frac{d_{w}^{4}G}{8D_{w}^{3}N_{a}} \end{array}$$ (8)

Assuming the spring starts at rest such that all the adjacent wires are wrapped to be touching with no loading, the rest length of the spring/windings on the sounding length of the string will be *l*_0_ = *N*_*a*_ *d*_*w*_ and the spring force on stretching this portion of the spring/windings to increase the length by Δ*l* will be

$$\begin{array}{c} T_{w}=k\Delta l=\frac{\Delta l}{l_{0}}\left(\frac{d_{w}^{5}G}{8D_{w}^{3}}\right)=\frac{\Delta l}{l_{0}}A_{w}\left(\frac{G}{2\pi }\right){\left(\frac{d_{w}}{D_{w}}\right)}^{3} \end{array}$$ (9)

where *A*_*w*_ = *π*(*d*_*w*_/2)^2^ is the cross-sectional area of the wire used in the winding. The most common choice of material in electric guitar strings is steel, and the winding is often nickel plated steel. In order to approximate typical values we will assume steel music wire construction [13] such that the Young’s modulus is *E* = 207 GPa and the modulus of rigidity is *G* = 79.3 GPa. Remembering that *D*_*w*_ is the mean diameter of the winding around the axis, we may define the core diameter as *d*_*core*_ = *D*_*w*_ − *d*_*w*_. In practice the core diameter is larger than the diameter of the wire in the winding, so that *d*_*w*_ < *d*_*core*_ and thus *d*_*w*_/*D*_*w*_ < 1/2, impling that

$$\begin{array}{c} T_{w}<\frac{\Delta l}{l_{0}}A_{w}\times 1.58\;\text{GPa}. \end{array}$$ (10)

The corresponding tension for the core would be given by Eq (1) as

$$\begin{array}{c} T_{core}=\frac{\Delta l}{l_{0}}A_{core}\times 207\;\text{GPa}. \end{array}$$ (11)

This proves that the tension contributed by the winding is less than one percent of the tension in the core and may be assumed to be negligible in most practical applications.

It is possible to construct a spring that is pretensioned (for instance through rotating the wire during cooling) and theoretically this could give the winding a stronger contribution to the overall tension force but doing so would reduce the benefits of using wound strings.

### Mass per unit length of wound strings

Defining the total mass per unit length of wound strings as *μ*_*cw*_, this will equal the sum of the mass per unit lengths of the core, *μ*_*core*_ and the mass per unit length of the windings, *μ*_*w*_, giving:

$$\begin{array}{c} \mu_{cw}=\mu_{core}+\mu_{w}=\tau \mu_{core}, \end{array}$$ (12)

where the factor *τ* is the mass per unit length of the string divided by the mass per unit length of the core. Expressing the mass per unit length of the core after stretching as

$$\begin{array}{c} \mu_{core}=\rho_{core}A_{core}\frac{l_{0}}{l_{f}}, \end{array}$$ (13)

where *ρ*_*core*_ is the density of the core when un-stretched and the cross-sectional area of the core when un-stretched is

$$\begin{array}{c} A_{core}=\gamma_{core}{\left(\frac{d_{core}}{2}\right)}^{2}, \end{array}$$ (14)

where *d*_*core*_ is the maximum core width and *γ*_*core*_ is a factor which depends on the cross-sectional shape of the core. For a cylindrical core, *d*_*core*_ is the core diameter and for hexagonal cross-section cores, *d*_*core*_ is defined as the distance between opposite points on the hexagon. The factor *γ*_*core*_ is given by:

$$\begin{array}{c} \gamma_{core}=\left\{ \begin{array}{cc} \frac{3\sqrt{3}}{2}, & \text{Hexagonal}\;\text{cross-section}\;\text{core}\\ \pi , & \text{Circular}\;\text{cross-section}\;\text{core.} \end{array}\right. \end{array}$$ (15)

Remembering that *D*_*w*_ is the diameter of the centre of the winding with respect to the axis of the core, the mass per unit length of the spring/windings (after stretching), *μ*_*w*_, is derived using the mass of a helical spring [13] to give:

$$\begin{array}{c} \mu_{w}=\frac{l_{0}}{l_{f}}\rho_{w}\frac{A_{w}\pi D_{w}N_{a}}{l_{0}}\approx \frac{l_{0}}{l_{f}}\rho_{w}A_{w}\left(\frac{\pi D_{w}}{d_{w}}\right) \end{array}$$ (16)

where *l*_0_ ≈ *N*_*a*_ *d*_*w*_ and again *A*_*w*_ = *π*(*d*_*w*_/2)^2^ is the cross-sectional area of the wire used in the winding, allowing the overall mass per unit length of the wound string to be given as:

$$\begin{array}{c} \mu_{cw}=\frac{l_{0}}{l_{f}}\left(\rho_{core}A_{core}+\rho_{w}A_{w}\left(\frac{\pi D_{w}}{d_{w}}\right)\right). \end{array}$$ (17)

Expressing this in terms of the maximum core width, *d*_*core*_, and using *D*_*w*_ = *d*_*core*_ + *d*_*w*_ gives:

$$\begin{array}{c} \mu_{cw}=\frac{l_{0}}{l_{f}}\tau \rho_{core}A_{core}, \end{array}$$ (18)

where the factor *τ* is given by

$$\begin{array}{c} \tau =(1+\left(\frac{\rho_{w}}{\rho_{core}}\right)\left(\frac{\pi^{2}}{\gamma_{core}}\right)\left(\left(\frac{d_{w}}{d_{core}}\right)+{\left(\frac{d_{w}}{d_{core}}\right)}^{2}\right)). \end{array}$$ (19)

This ratio of the mass per unit length of the string in relation to that of the core will be in the range *τ* > 1 for wound strings. The above equations are also valid for unwound strings of circular cross-section by substituting *d*_*w*_ = 0 and *γ*_*core*_ = *π* as then *τ* = 1. Hexagonal cores wound with a circular cross-section winding have $\gamma_{core}=\frac{3\sqrt{3}}{2}$. Issues such as the deviation of the core and winding from idealised shapes mean that, in practice, it is more accurate to establish an accurate value of *τ* for a string destructively, based on cutting a length of string, unwrapping the winding from the core and measuring the ratio:

$$\begin{array}{c} \tau =\frac{m_{core}+m_{w}}{m_{core}}, \end{array}$$ (20)

where *m*_*core*_ is the mass of the core and *m*_*core*_ + *m*_*w*_ is the total mass of the same length of string.

### Sounding frequency and strain on wound strings

Assuming that the tension supported by the winding is negligible, we may return to Eq (1) considering just the core such that:

$$\begin{array}{c} T_{f}\approx T_{core}=E_{core}A_{core}\frac{\Delta l}{l_{0}}. \end{array}$$ (21)

Now using this to calculate the frequency using Eqs (2) and (18) gives:

$$\begin{array}{c} \nu_{f}=\frac{1}{2l_{f}}\sqrt{\frac{E_{core}\frac{\Delta l}{l_{0}}}{\rho_{core}\tau \frac{l_{0}}{l_{f}}}} \end{array}$$ (22)

or

$$\begin{array}{c} \nu_{f}=\frac{1}{2l_{f}}\sqrt{\frac{E_{core}\left(\frac{\Delta l}{l_{f}}\right)}{\rho_{core}\tau {\left(1-\frac{\Delta l}{l_{f}}\right)}^{2}}}. \end{array}$$ (23)

Squaring both sides and solving the resulting quadratic equation gives the engineering strain as:

$$\begin{array}{c} \frac{\Delta l}{l_{f}}=1+\frac{E_{core}}{2\rho_{core}\tau {\left(2l_{f}\nu_{f}\right)}^{2}}\left(1-\sqrt{1+\frac{4\rho_{core}\tau {\left(2l_{f}\nu_{f}\right)}^{2}}{E_{core}}}\right) \end{array}$$ (24)

The binomial expansion of this for Δ*l* ≪ *l*_*f*_ gives:

$$\begin{array}{c} \frac{\Delta l}{l_{f}}=\left(\frac{\rho_{core}\tau {\left(2l_{f}\nu_{f}\right)}^{2}}{E_{core}}\right)-2{\left(\frac{\rho_{core}\tau {\left(2l_{f}\nu_{f}\right)}^{2}}{E_{core}}\right)}^{2}+5{\left(\frac{\rho_{core}\tau {\left(2l_{f}\nu_{f}\right)}^{2}}{E_{core}}\right)}^{3}-\ldots \end{array}$$ (25)

## Theoretical tremolo arm pitch deviations

Consider again a string initially tuned up to sounding length *l*_*f*_ = *l*_0_ + Δ*l* and fundamental frequency *ν*_*f*_ when no pressure is applied to the tremolo arm. Suppose that the sounding length the string is then changed by a distance *δ* using the tremolo arm to give a new sounding length of *l*_*t*_ = *l*_0_ + Δ*l* + *δ*. It is important to note that, in a standard (non-locking) nut design, the string will slip across the nut to approximately equalise the tension either side of the nut. This is clear from the fact that pressing the string behind the nut to increase the tension there also produces a corresponding pitch increase in the main portion of the string (and the main portion of the string returns to the original pitch when the pressure behind the nut is released, assuming the nut is in good condition). This means that, when the sounding length changes by *δ*, a fraction of that length change is taken up by the portion of string behind the nut. The portion of the string responsible for the original sounding length (which is responsible for calculating changes in tension and density) actually slides to occupy a different length *l*_*t*_ = *l*_0_ + Δ*l* + *δ*_*c*_ where:

$$\begin{array}{c} \delta_{c}=\delta \frac{l_{f}}{l_{f}+l_{nut}}, \end{array}$$ (26)

with *l*_*nut*_ being the length of string behind the nut. The resulting fundamental frequency, labelled *ν*_*t*_, will change (using Eq (22)) to give:

$$\begin{array}{c} \nu_{t}=\frac{1}{2(l_{f}+\delta )}\sqrt{\frac{E_{core}\frac{\Delta l+\delta_{c}}{l_{0}}}{\rho_{core}\tau \frac{l_{0}}{l_{f}+\delta_{c}}}} \end{array}$$ (27)

hence the sounding frequency changes by the ratio:

$$\begin{array}{c} \frac{\nu_{t}}{\nu_{f}}=\left(\frac{1}{1+\frac{\delta }{l_{f}}}\right)\sqrt{\left(1+\frac{\delta_{c}}{\Delta l}\right)\left(1+\frac{\delta_{c}}{l_{f}}\right)}. \end{array}$$ (28)

Since *δ* ≪ *l*_*f*_ and hence *δ*_*c*_ ≪ *l*_*f*_, the ratio *δ*_*c*_/Δ*l* dominates the resulting equation giving a first approximation of $\nu_{t}/\nu_{f}\approx \sqrt{1+(\delta_{c}/\Delta l)}$. The change in cents (where a cent is defined as one percent of a musical interval of a semitone) is given by [12]:

$$\begin{array}{c} {\text{interval}}_{f\to t}\left(\text{cents}\right)=1200\log_{2}\left(\frac{\nu_{t}}{\nu_{f}}\right) \end{array}$$ (29)

Returning to Fig 1, when the tremolo arm rotates by an angle of *θ* around the pivot axis then the total change in sounding length of the string will be given by:

$$\begin{array}{c} \delta =2R_{a}\sin\left(\theta /2\right)\sin\left(\phi -\left(\theta /2\right)\right), \end{array}$$ (30)

where (*R*_*a*_, *ϕ*) and (*R*_*a*_, *ϕ* − *θ*) are the initial and final positions of the point of contact between the string and the saddle from the pivot in polar coordinates. Due to slippage at the nut, the length change responsible for changes in tension and mass per unit length is then:

$$\begin{array}{c} \delta_{c}=2R_{a}\sin\left(\theta /2\right)\sin\left(\phi -\left(\theta /2\right)\right)\left(\frac{l_{f}}{l_{f}+l_{nut}}\right). \end{array}$$ (31)

## Predicted and measured tremolo arm pitch deviations

A common setup for a Stratocaster style tremolo system is to “float the bridge” (as shown in the original Fender patent drawings [3], by setting the height of the bridge plate block above the body when tensioned to standard tuning by adjustment of the tremolo screws and tuners. This is commonly done such that the G string goes a minor third sharp when the tremolo arm is pulled fully up so that it is touching the body (such that ${\text{interval}}_{f\to t}^{\left(3\right)}\approx 300$ cents) as this generally leads to an approximation to musically useful intervals of a whole tone for the B string (${\text{interval}}_{f\to t}^{\left(2\right)}\approx 200$ cents) and a semitone for the high E string (${\text{interval}}_{f\to t}^{\left(1\right)}\approx 100$ cents). This occurs because the change in length of the string required to bring it up to tune in the first place, Δ*l*, is larger for the higher pitched strings and hence they are less sensitive to the similar values of string length change *δ* in Eq (28).

An American made Fender Stratocaster was used for the measurements presented here (Oiled Ash 10 for 15 limited edition). This instrument has the American standard two post Fender Stratocaster tremolo system with a 9.5 inch radius neck (as on the American Standard Stratocaster) and was modified with Graph Tech String Saver Classics saddles and Schaller F-Series Locking Tuners. Adjustment of the saddle positions and tremolo claw screws was performed to obtain typical string heights, good intonation and 300 ± 6 cents of pitch increase for a full pull up. The measured (and where necessary assumed) parameters for the open string playing frequencies in standard tuning and values for the relevant distances are given in Table 1. The unwound (E_4_, B_3_ and G_3_) strings were assumed to be constructed of plain steel. The wound (D_3_, A_2_ and E_2_) strings featured in the initial experiments and calculations (D’Addario EXL120) feature hexagonal cross-section steel cores.

**Table 1:** String parameters for american fender stratocaster.

Measured						Assumed values	
Pitch	*ν*_*f*_(Hz)	*l*_*f*_(mm)	*l*_*nut*_ (mm)	*R*_*a*_(mm)	*ϕ*(rads)	*E*_*core*_(GPa)	*ρ*_*core*_(kg/m^3^)
*E*_4_	329.6	647.8	159.1	9.2	1.13	207	7860
*B*_3_	246.9	648.3	135.4	9.9	1.11	207	7860
*G*_3_	196.0	649.6	112.3	11.3	1.04	207	7860
*D*_3_	146.8	649.0	87.9	11.0	1.09	207	7860
*A*_2_	110.0	649.6	64.5	10.7	1.01	207	7860
*E*_2_	82.4	650.1	41.1	10.6	0.95	207	7860

Sounding fundamental frequency *ν*_*f*_ given to 4 significant figures. Sounding length *l*_*f*_ is ±1 mm but with differences between sounding lengths accurate to 0.1 mm. Length of string between nut and tuning peg is *l*_*nut*_ ± 0.5 mm. Distance from pivot to centre of string at point of contact with saddle is *R*_*a*_ ± 0.1 mm. Angle of point of contact with saddle from pivot with respect to body is *ϕ* ± 0.01 rads. Density and Young’s modulus for steel core are from [13].

After all tests were complete, the strings were removed from the guitar and cut at the positions were they showed permanent bends from meeting the tuners and saddles (so cut to length *l*_*f*_ + *l*_*nut*_). The winding was then pulled from the core by hand. While pulling the winding, the core rotated rapidly around the winding, exposing a section of core, typically around 10 cm in length on each pull. The exposed section of core was then cut and retained for weighing and the process repeated until all the core sections and windings were separated for weighing. The masses of the cores and windings were then measured using laboratory scales (Unimatic CL41) and Eq (20) used to determined the value of *τ* for each string.

The quantities listed in Table 1 were then used to calculate the values given in Table 2. The theoretical predictions of the pitch intervals obtained were then compared to those measured experimentally. Experimental values were obtained by plugging the electrical output from the guitar into a RME Fireface UFX sound card using a standard jack cable. The RME Fireface was connected (using a USB cable) to a MacBook Pro laptop computer running Apple Logic Pro X software. A track was then set up with input monitoring and an instance of the software’s built in Tuner was enabled to monitor the sounding pitch of the guitar when plucked by the author. The guitar tuning process involved:

1. Plucking the strings lightly, one at a time and turning the tuning pegs until the tuner in Logic showed the strings as being in tune to within 2 cents.
2. Performing a full pull up (pulling the tremolo arm away from the body) so that the (initially floating) bridge plate contacted the body and plucking the string lightly, noting the pitch in cents (in real time) on the Logic tuner before releasing the tremolo arm.
3. Plucking the strings lightly and noting the tuning of the strings after the tremolo arm is released.
4. If the final pitch of the string is more than 2 cents out of tune then the tuning and measurement process is repeated from step 1.

In practice it takes a small number of repeated pull ups and re-tunings before stable operation with accurately reproducible results is achieved on instruments with a standard (non-locking) Stratocaster style bridges.

**Table 2:** Theoretically predicted versus experimentally measured pitch deviation for a tremolo arm pull up by *θ* = 0.085 radians assuming dimensions and variables taken from Table 1 with D’Addario EXL120 “Nickel Wound” Super Light 9-42 strings (round wound with nickel plated steel).

Theoretical							Experimental
Starting Pitch	Δ*l*/*l*_*f*_ (%)	Δ*l* (mm)	*δ* (mm)	*δ*_*c*_ (mm)	*τ*	interval_*f* → *t*_ (cents)	interval_*f* → *t*_ (cents)
*E*_4_	0.683	4.43	0.692	0.556	1	101	105
*B*_3_	0.386	2.50	0.737	0.610	1	187	185
*G*_3_	0.245	1.59	0.807	0.688	1	310	300
*D*_3_	0.377	2.45	0.810	0.713	2.76	220	222
*A*_2_	0.277	1.80	0.749	0.681	3.59	277	285
*E*_2_	0.207	1.34	0.710	0.668	4.76	348	356

The ratio of total string mass to core mass, *τ*, was taken from Eq (20) while the expected engineering strains, Δ*l*/*l*_*f*_, were calculated using Eq (24). Eqs (30) and (31) were used to calculate the change in the sounding length, *δ*, and the change in length of the original sounding length allowing for slippage at the nut, *δ*_*c*_, based the measured value for the tremolo arm pull angle, *θ* = 0.085 radians = 4.9°. Values of the pitch deviation interval in cents were then obtained by placing values obtained using Eq (28) into Eq (29). Experimental data was measured on the guitar noting pitch intervals on the Logic Pro X tuner (with the display giving results that were consistent to plus or minus 6 cents). All values are quoted to 3 significant figures.

Low dynamic level plucks were performed by the author to check the tuning of notes in cents in order to avoid downward pitch drifts of over 10 cents observed during the sustain of some louder notes, a nonlinearity resulting from increased displacements causing increased tensions. The neck was supported at the heel by the left hand (at the connection between the neck and the body). It should be noted that supporting, pressing or pulling the neck with the left hand can lead to additional sources of pitch changes. All strings other than the one being plucked were damped lightly with the left hand fingers in order to prevent sympathetic resonance causing instability in the tuner output.

It is clear that there is reasonable agreement between experiment and theory for the pitch interval produced by pulling the tremolo arm fully up. The pitch intervals for the unwound strings are reasonable close to the 100 cents, 200 cents and 300 cent values both in the theoretical predictions and in the experimental measurements. Errors in determining the angle of tremolo arm movement would give a similar error for different strings so the small under-prediction in the number of cents on pull up for the high E (E_4_) string in comparison to the error on the G string cannot be explained by this.

It is worth noting from Table 2 that engineering strains of 0.2% are exceeded on all strings. The high E string (E_4_) has the largest engineering strain of almost 0.7%. Such strains ensures that new strings exceed the linear elastic region of the stress-strain graph for steel (see [9]) and are observed to deform within seconds of first being brought up to pitch (increasing the rest length and dropping the sounding pitch) and thus requiring further stretching using the tuner. This process continues until the material has undergone sufficient stretching to be essentially stable (and this process of “stretching” new strings is well known to players). It may be assumed that this process is a form of strain hardening or work hardening [14] (and the material in the string becomes saturated in terms of the generation of the internal dislocations that can generate permanent changes in dimensions).

The Ultimate Tensile Strength (maximum stress before breakage) of the type Ia or IIa steel used in strings is typically around 2.1—2.5 GPa [9] and the stress on the high E string will be *E*_*core*_Δ*l*/*l*_*f*_ ≈ 1.41 GPa meaning that this string is operating at around 60 ± 10% of the Ultimate Tensile Strength (even before any tremolo arm movement). With the tremolo arm movement increasing the pitch as described, the engineering strain increases to approximately (Δ*l* + *δ*_*c*_)/*l*_*f*_ giving a stress of *E*(Δ*l* + *δ*_*c*_)/*l*_*f*_ or 70 ± 10% of the UTS for the high E string (E_4_). Considering the low E string (with the lowest stress on the core) the resulting stress at rest is 0.43 GPa meaning that this string is operating at around 19% of the UTS (and operates at around 28 ± 3% for a full pull up). It is worth noting that it has been stated that the best tone available for stress corresponding to around 40% to 70% of the Ultimate Tensile Strength [9].

**Fig 2:**
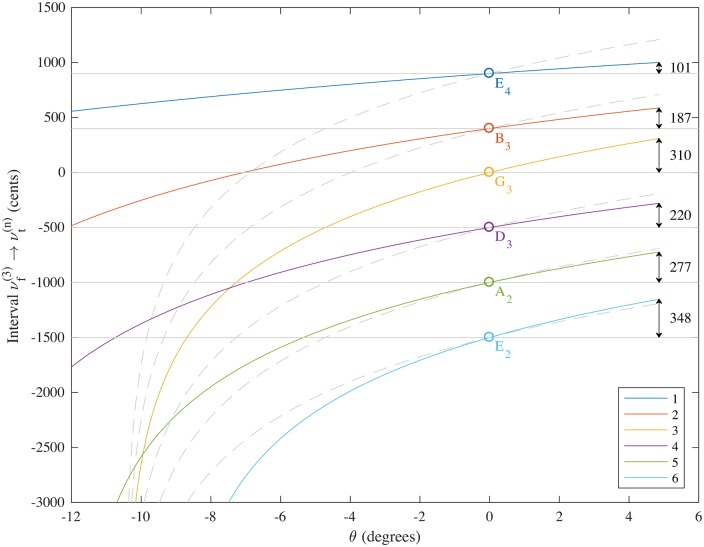
Theoretical pitch deviations (in cents with respect to the pitch G_3_) as a function of *θ* (in degrees) for Fender Stratocaster with D’Addario EXL120 Super Light 9-42 strings. Dimensions and variables are taken from Table 1. Equations used were Eqs (20), (24), (30), (31), (28) and (32). Dashed grey lines on the figure show the interval predicted for the 3rd string transposed by a constant number of cents corresponding to the relative tuning of each string when *θ* = 0.

Theoretical pitch deviations for a wider range of values of *θ* are shown in Fig 2 for the six open strings, all expressed in cents relative to the pitch G_3_. The maximum *θ* value plotted corresponds to a full pull up of *θ* = 0.085 rads = 4.9° to 2 significant figures. Negative values of *θ* show decreasing sounding pitches for the strings with the 6th string (original sounding pitch low E (E_2_)) going completely slack first (due to its large core diameter) as *θ* becomes more negative. Again the dimensions and variables are taken from Table 1 corresponding to the Fender Stratocaster with D’Addario EXL120 Super Light 9-42 strings. Cent values were calculated using the formula:

$$\begin{array}{c} {\text{interval}}_{\nu_{f}^{\left(3\right)}\to \nu_{t}^{\left(n\right)}}=1200\log_{2}\left(\frac{\nu_{t}^{\left(n\right)}}{\nu_{f}^{\left(3\right)}}\right) \end{array}$$ (32)

where *n* is the number of the string (strings 1, 2, 3, 4, 5 and 6 have the sounding pitches E_4_, B_3_, G_3_, D_3_, A_2_ and E_2_ respectively when the tremolo in the rest position (*θ* = 0)). Note that the relative tunings for the 1st, 2nd, 4th, 5th and 6th strings with respect to the 3rd string correspond to the standard tuning intervals of +900, +400, -500, -1000 and -1500 cents respectively. Dashed grey lines on the figure show the tuning of the 3rd string transposed through these numbers of cents so that the relative tuning of the strings is clear. It should be noted that the 3rd and 5th strings are closest to retaining an in tune relationship for this set of strings while the pitch deviations of the 1st and 2nd strings are much smaller than that required for remaining in tune with tremolo arm use (as dictated by their unwound construction).

## Theoretical control parameters for pitch deviations and experimental verification

The theoretical sounding frequencies implied by the equations above may be used to assess the effect of various adjustments in the parameters which could be typically observed in design and setting up electric guitars and their strings. Table 3 displays typical results for these theoretical predictions.

**Table 3:** Theoretically predicted pitch deviation tabulated for a tremolo arm pull up by *θ* = 0.085 radians assuming dimensions and variables taken from Table 1.

Pitch	*R*_*a*_ (mm)	*l*_*nut*_ (mm)	*d*_*w*_/*d*_*core*_	*ρ*_*core*_ (kg/m^3^)	*ρ*_*w*_ (kg/m^3^)	*τ*	interval_*f* → *t*_ (cents)	Comment
*E*_4_	9.2	159.1	0	7860	0	1	101	Standard
*B*_3_	9.9	135.4	0	7860	0	1	187	Standard
*G*_3_	11.3	112.3	0	7860	0	1	310	Standard
*D*_3_	11.0	87.9	0.342	7860	7942	2.76	220	Standard
*A*_2_	10.7	64.5	0.462	7860	7942	3.59	277	Standard
*E*_2_	10.6	41.1	0.609	7860	7942	4.76	348	Standard
*E*_4_	9.7	159.1	0	7860	0	1	106	Raised Saddle
*B*_3_	10.4	135.4	0	7860	0	1	196	Raised Saddle
*G*_3_	11.8	112.3	0	7860	0	1	321	Raised Saddle
*D*_3_	11.5	87.9	0.342	7860	7942	2.76	228	Raised Saddle
*A*_2_	11.2	64.5	0.462	7860	7942	3.59	288	Raised Saddle
*E*_2_	11.1	41.1	0.609	7860	7942	4.76	362	Raised Saddle
*E*_4_	9.2	41.1	0	7860	0	1	118	Reverse Neck
*B*_3_	9.9	64.5	0	7860	0	1	204	Reverse Neck
*G*_3_	11.3	87.9	0	7860	0	1	318	Reverse Neck
*D*_3_	11.0	112.3	0.342	7860	7942	2.76	213	Reverse Neck
*A*_2_	10.7	135.4	0.462	7860	7942	3.59	255	Reverse Neck
*E*_2_	10.6	159.1	0.609	7860	7942	4.76	305	Reverse Neck
*E*_4_	9.2	0.0	0	7860	0	1	125	Locking Nut
*B*_3_	9.9	0.0	0	7860	0	1	222	Locking Nut
*G*_3_	11.3	0.0	0	7860	0	1	354	Locking Nut
*D*_3_	11.0	0.0	0.342	7860	7942	2.76	246	Locking Nut
*A*_2_	10.7	0.0	0.462	7860	7942	3.59	300	Locking Nut
*E*_2_	10.6	0.0	0.609	7860	7942	4.76	366	Locking Nut
*D*_3_	11.0	87.9	0.342	7860	8885	2.97	206	“Pure Nickel”
*A*_2_	10.7	64.5	0.462	7860	8885	3.9	258	“Pure Nickel”
*E*_2_	10.6	41.1	0.609	7860	8885	5.21	323	“Pure Nickel”
*D*_3_	11.0	87.9	0.479	7860	7942	3.72	168	Narrower Core
*A*_2_	10.7	64.5	0.647	7860	7942	5.09	204	Narrower Core
*E*_2_	10.6	41.1	0.853	7860	7942	7.06	249	Narrower Core
*D*_3_	11.0	87.9	0.205	7860	7942	1.95	297	Wider Core
*A*_2_	10.7	64.5	0.277	7860	7942	2.36	392	Wider Core
*E*_2_	10.6	41.1	0.365	7860	7942	2.92	513	Wider Core
*D*_2_	10.6	41.1	0.609	7860	7942	4.76	419	Drop D

The first six rows repeat values from Tables 1 and 2 while the later rows illustrate the theoretical variation in pitch intervals produced by adjustments of control parameters. The values of *d*_*w*_/*d*_*core*_ were chosen to be consistent with the measured values of *τ*, where standard nickel plated steel windings are assumed to be 92% steel with 8% nickel plating with the density of nickel (8885 kg/m^3^). The effect of using interchanging windings made of pure nickel of the same diameter instead of nickel plated steel is also shown in the row labelled “Pure Nickel”. Equations used were Eqs (19), (24), (30), (31), (28) and (29) with the results computed using a MATLAB script. Values which are greater than or lower than standard are given background colors of yellow and light grey respectively.

### The effect of saddle height

An adjustment of the distance between the point of string contact with the saddle and the pivot, *R*_*a*_, by a distance of 0.5mm (as would happen if the height of the saddle on screws was adjusted by around *R*_*a*_/sin *ϕ* ≈ 0.6 mm) produces a slight increase in the sensitivity (the column labelled interval_*f* → *t*_ giving the number of cents change with tremolo arm use) of between 3.6% and 5.0%. Such height differences are typically observed between instruments with a different fretboard radius.

Experimental verification of the effect of saddle height was conducted by increasing the saddle height on for the Fender Stratocaster’s high E (E_4_) string by 0.6 mm (and retuning the strings to standard pitch). The result was that the interval produced by a full pull up changed from 105 ± 6 cents to 111 ± 6 cents, validating the theoretical prediction from Table 3. This control parameter is useful for fine tuning the relative intervals if desired.

### The effect of varying the length of string behind the nut through use of a reverse headstock or a locking nut

Using a reverse neck (also known as a reverse headstock) guitar, where the longest distance behind the nut, *l*_*nut*_, on the low E (E_2_) string instead of the high E (E_4_) string, produces a relatively large reduction in the number of cents of change with tremolo arm use (of roughly 14%) for the low E string.

Using a locking nut raises the pitch sensitivity of all strings, with the high E string (E_4_) being adjusted by the greatest fraction (roughly 23%). Experimental verification of the effect of a locking nut was conducted using a Floyd Rose locking nut equipped guitar (Washburn KC-40V). All three bolts that clamp the strings at the nut were first removed (allowing the string to slide over the nut area which was lubricated with a commercially available lubricant called “Big Bends Nut Sauce”) and the guitar tuned to standard tuning. A block was then inserted into the tremolo cavity to give 300 ± 6 cents increase in pitch for the G string. The high E (E_4_) string was observed to sharpen by 113 ± 6 cents due to the insertion of this block. With the process repeated with the high E string clamped at the nut using one of the locking nut bolts and leaving the G string unclamped. The same block was then inserted to give 300 ± 6 cents for the unclamped G string, and the resulting interval for the high E (E_4_) string was 137 ± 6 cents, an increase of 21 ± 11%. While this is a different instrument to that used for the theoretical predictions from Table 3 (with larger values of *R*_*a*_ for instance), the effect is clearly measurable and of the correct order.

Various alternative headstock designs are also popular, including the traditional design featuring three tuners on each side of the headstock as featured on most guitars by the manufacturer Gibson. The altered lengths of string behind the nut (*l*_*nut*_) for such designs introduce adjustments to the relative intervals obtained under tremolo arm use and these adjustments are similar in magnitude to the difference between the Fender and Locking Nut designs previously discussed. Most alternative bridge/tailpiece designs such as the Bigsby vibrato tailpiece are expected to have similar relative pitch intervals to the Fender design because they operate by simultaneously introducing similar length changes to the different strings (though they typically have smaller ranges of maximum pitch deviation). A full treatment of this topic is beyond the scope of the current paper.

### The effect of gauge, tension and alternative tunings

In deriving the equations used in this model, it was noted that the engineering strain required to tune up a string from Eq (24) doesn’t depend on the overall gauge or total diameter of the string. In turn this means that the interval produced by a given tremolo arm movement doesn’t depend on the gauge for a given tuning (although the ratio of core to winding mass does have a significant impact in wound strings). The effect of the string gauge was investigated experimentally by means of removing the unwound D’Addario 0.016 inch diameter steel G_3_ string and replacing it with an unwound Ernie Ball Custom Gauge 0.014 inch diameter steel string G_3_ string (giving a lower than normal tension for that string). It was found that by adjustment of the tremolo claw screws together with retuning the strings to standard pitch enabled the same pitch intervals to be obtained from the “Experimental” column of Table 1 within the experimental error of around 6 cents. Different gauges are still significant from the player’s perspective as this adjusts the overall tension for a given tuning (and the thus alters the forces the player must apply for a given effect).

In practice setting up an instrument for a new gauge (including truss rod adjustment to counteract string tension changes) may lead to small, indirect changes in relative tuning with tremolo arm use in addition to the expected changes in force required to produce a given distance of tremolo arm movement.

Changing to an alternative tuning will have a big impact on the pitch changes obtained by tremolo arm use because the engineering strain is roughly proportional to the sounding frequency squared for a given string construction (from Eq (25)). Table 3 illustrates this with the example of the lowest pitch string being detuned down a whole tone to D_2_ (as in various popular alternative tunings including drop D). The change in cents with tremolo arm usage increases from 348 cents to 419 cents. This illustrates the fact that detuned (flattened) strings are oversensitive to player’s control changes in general.

### The effect of core to winding mass ratio

The most dramatic result from the theoretical analysis is that changing the ratio of the diameters of the winding and core results in large change in sensitivity to tremolo arm use. As an example, increasing the ratio *d*_*w*_/*d*_*core*_ by 40% from 0.609 to 0.853 results in a 28% reduction in the interval produced by tremolo arm movement for the low E (E_2_) string for instance. In practice this can be achieved by using a narrow core and wider winding to obtain a string with a similar tension at pitch. Conversely, increasing the diameter of the core and reducing the diameter of the winding in the D_3_ string helps to equalise the sensitivity with the G_3_ string. This is simulated in the table by showing the theoretical effect of decreasing *d*_*w*_/*d*_*core*_ by 40%, increasing the interval for a full pull up by 35%.

Using pure nickel windings (instead of nickel plated steel) on steel cores increases the mass in the windings and this reduces the sensitivity of the wound strings, again helping to equalise the low E (E_2_) and G strings but also reducing the sensitivity of the D string. It should be noted that strings designed with pure nickel windings will often feature different diameters for core and windings and the sensitivity of the strings is heavily dependent on this parameter (as discussed below).

Experimental verification of the effect of different core to winding mass ratios was conducted by replacing the strings with D’Addario EPN120 “Pure Nickel” Super Light 9-41 strings (round wound with pure nickel). While labelled as “Pure Nickel”, the unwound strings and the cores of all the wound strings in these sets (as with all electric guitar string sets known to the author) are made of steel due to the low Ultimate Tensile Strength of nickel (maximum values being less than 1.2 GPa [15] so roughly half that of typical values for the steel used in strings). The windings are, as the name suggests, made of pure nickel and the larger density of this metal would be expected, if the strings core and winding diameter ratios were unchanged, to give a larger total mass to core mass ratio (*τ*) and hence a larger engineering stain at pitch and therefore a lower sensitivity to length changes with tremolo use.

With the “Pure Nickel” strings fitted, it was necessary to tighten the tremolo claw screws a fraction of a turn (and retune) to counteract a slightly larger tension of the strings at pitch so that the 3rd (G_3_) string again completed 300 cents of pitch increase on a full pull up (again giving *θ* = 0.085 radians for a full pull up). While the intervals produced by the unwound strings were unchanged, those of the wound strings changed to those given in Table 4. The wound strings were then cut at the points of contact with the tuning posts and saddles, the windings pulled off the cores and the masses measured on mg accurate scales to establish the ratio of total mass to core mass, *τ*, for the strings, accurate to three significant figures. This enabled the theoretical calculations for a full pull up with *θ* = 0.085 radians to be added to Table 4 for comparison.

**Table 4:** Theoretically predicted versus experimentally measured pitch deviation for a tremolo arm pull up by *θ* = 0.085 radians assuming dimensions and variables taken from Table 1 with D’Addario EPN120 “Pure Nickel” Super Light 9-41 strings (round wound with pure nickel).

Theoretical							Experimental
Starting Pitch	Δ*l*/*l*_*f*_ (%)	Δ*l* (mm)	*δ* (mm)	*δ*_*c*_ (mm)	*τ*	interval_*f* → *t*_ (cents)	interval_*f* → *t*_ (cents)
*D*_3_	0.331	2.15	0.810	0.713	2.41	247	255
*A*_2_	0.287	1.86	0.749	0.681	3.72	269	275
*E*_2_	0.220	1.43	0.710	0.668	5.08	330	342

Dimensions and variables are taken from Table 1. The values of *τ* were taken from destructive testing of the strings and utilising Eq (20). Other equations used for the theoretical calculations were Eqs (24), (30), (31), (28) and (29). Experimental data was measured on the guitar noting pitch intervals on the Logic Pro X tuner (with the display giving results that were consistent to plus or minus 6 cents). All values are to 3 significant figures.

The core of the 4th (D_3_) string was significantly wider and the winding significantly narrower for the EPN120 “Pure Nickel” set and this lead to a noticeably lower value of *τ* and hence a larger pitch deviation on tremolo arm use in comparison to the standard EXL120 set for this string. Experimental data differs from the theoretical prediction for this string by only 8 cents, validating the theoretical prediction of the intervals produced by tremolo arm movement. In this case, the increase in core diameter and decrease in winding diameter for the D_3_ string reverses the result which would expected if only the volumetric density of the winding had changed (as shown in Table 3).

The cores of the 5th and 6th strings of the EPN120 set were close to identical to those of the EXL120 set while the corresponding diameter for the windings was slightly reduced in comparison to the EXL120 set, compensating for the increased density and leading to a similar values for *τ* and pitch interval changes. Experimental and theoretical results differ by up to 12 cents and the reason for this discrepancy (of 3.6% in the overall interval value) is likely to be principally due to the effect of the finite thickness of the strings.

## Custom wound strings with close to equal pitch deviations

Tensions for different strings in the same set tend to be within about 50% of one another in order to prevent warping of the neck and for a consistent playing experience. Different wound strings in the same set will therefore tend to have similar values for *T*_*f*_ ≈ *E*_*core*_ *A*_*core*_Δ*l*/*l*_0_ from Eq (21). To a rough first approximation they will have a very similar sensitivity to a given angle of tremolo movement if Δ*l*/*l*_0_ is the same for the different strings and this implies that they should share approximately the same cross-sectional area for the core, *A*_*core*_. This also implies that the strings would then have close to the same value for the stress *T*_*f*_/*A*_*core*_ meaning that the stress will be approximately the same fraction of the Ultimate Tensile Strength. A benefit of this is that the low E string may be prevented from having a stress below the range recommended in Jahnel [9] for optimum tone quality. From Eq (24) it is clear that for different strings constructed of the same core material, the value of $\tau \nu_{f}^{2}$ should be the approximately the same. Labelling the number of cents between the fundamental frequencies of the strings as “interval”, the ratio between the fundamental frequencies of two strings (before any tremolo arm force is applied) will be given by $\nu_{f}^{\left(m\right)}/\nu_{f}^{\left(n\right)}=2^{\text{interval}/1200}$. The result is that, for matched core diameters, tensions and sensitivities, the mass ratios for the strings should be related by:

$$\begin{array}{c} \tau^{\left(n\right)}\approx \tau^{\left(m\right)}2^{\text{interval}/600}. \end{array}$$ (33)

Consider the case of an unwound string (string number *m*) of diameter *d*_*core*_, and a wound string (string number *n*) of core diameter also equal to *d*_*core*_ but with windings constructed using wire of diameter $d_{w}^{\left(n\right)}$. This may be combined with Eq (19), which gives the mass ratio *τ*^(*n*)^ in terms of the core diameter, *d*_*core*_, and the diameter of the winding, $d_{w}^{\left(n\right)}$ to produce a quadratic in the ratio $d_{w}^{\left(n\right)}/d_{core}$ which may be solved to give:

$$\begin{array}{c} \frac{d_{w}^{\left(n\right)}}{d_{core}}\approx -\frac{1}{2}+\frac{1}{2}\sqrt{1+\left(\frac{4}{\beta }\right)\left(\left(2^{\text{interval}/600}\right)-1\right)}; \end{array}$$ (34)

where *β* = (*ρ*_*w*_/*ρ*_*core*_)(*π*^2^/*γ*_*core*_) and it should be noted that *τ*^(*m*)^ = 1 for the unwound string.

A set of strings (with cylindrical steel cores and nickel plated steel windings) was therefore designed that show close to equal pitch change intervals for the G_3_, D_3_, A_2_ and E_2_ strings. These were constructed by Newtone strings to the author’s own specification as given in Table 5. The values of *d*_*w*_ were increased where necessary (by up to 22%) in order to utilise available gauges of wire and to compensate for various approximations made during the derivation of Eq (34) (such as ignoring the fact that windings become oval during manufacturing, the effect of the different distances behind the nut, *l*_*nut*_, and the different positions of the saddles with respect to the pivot). The result is a set of strings with a significantly lower fraction of the mass in the windings (*τ* approximately 28% smaller) than usual on the D_3_ string, and a significantly greater fraction of mass in the windings (*τ* approximately 30% greater) than usual on the E_2_ string.

**Table 5:** Custom string set design parameters.

Pitch	*d*_*core*_ (inch)	*d*_*w*_ (inch)	*d*_*w*_/*d*_*core*_	*γ*_*core*_
E_4_	0.009	0	0	*π*
B_3_	0.011	0	0	*π*
G_3_	0.016	0	0	*π*
D_3_	0.016	0.004	0.250	*π*
A_2_	0.016	0.008	0.500	*π*
E_2_	0.016	0.014	0.875	*π*

The cylindrical cores for the 4th, 5th and 6th strings all match the diameter of the unwound (cylindrical) 3rd string. The winding diameters for the 4th, 5th and 6th strings are chosen to be an available diameter of wire close to that specified in Eq (34) such that the engineering strain, string stress and sensitivity to control changes are close equalling that of the unwound 3rd string when brought up to pitch.

**Fig 3:**
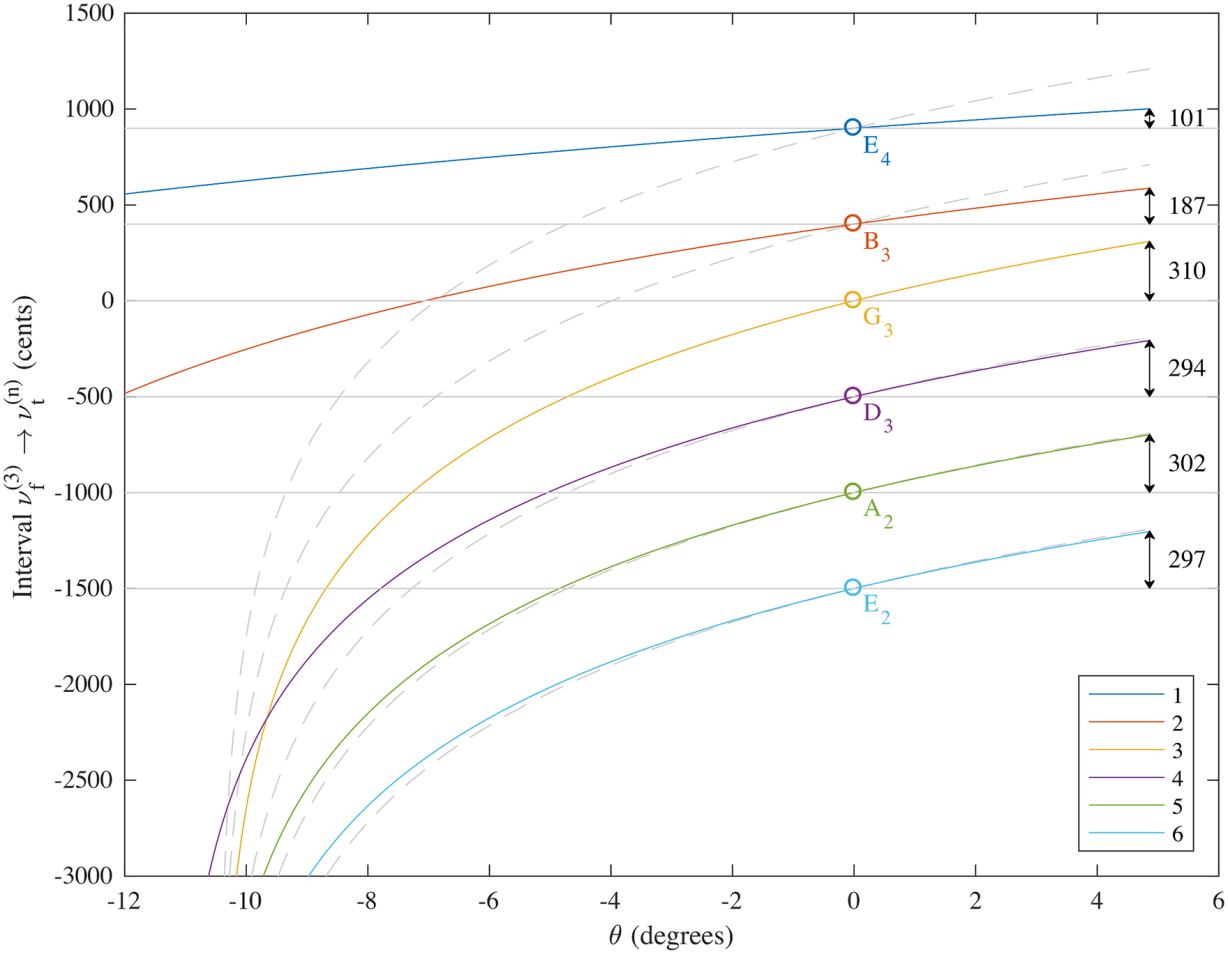
Theoretical pitch deviations (in cents with respect to the pitch G_3_) as a function of *θ* (in degrees) for Fender Stratocaster with custom strings made by Newtone Strings to the author’s specification. Dimensions and variables are taken from Table 1. Equations used were Eqs (20), (24), (30), (31), (28) and (32). Dashed grey lines on the figure show the interval predicted for the 3rd string transposed by a constant number of cents corresponding to the relative tuning of each string when *θ* = 0.

The theoretical pitch deviations for this set of strings is shown in Fig 3. It is clear that the predicted pitch deviations for the 3rd, 4th, 5th and 6th strings are much more similar for this custom string set, meaning that four note chords are predicted to remain close to being in tune over a much wider range of tremolo arm motion.

**Fig 4:**
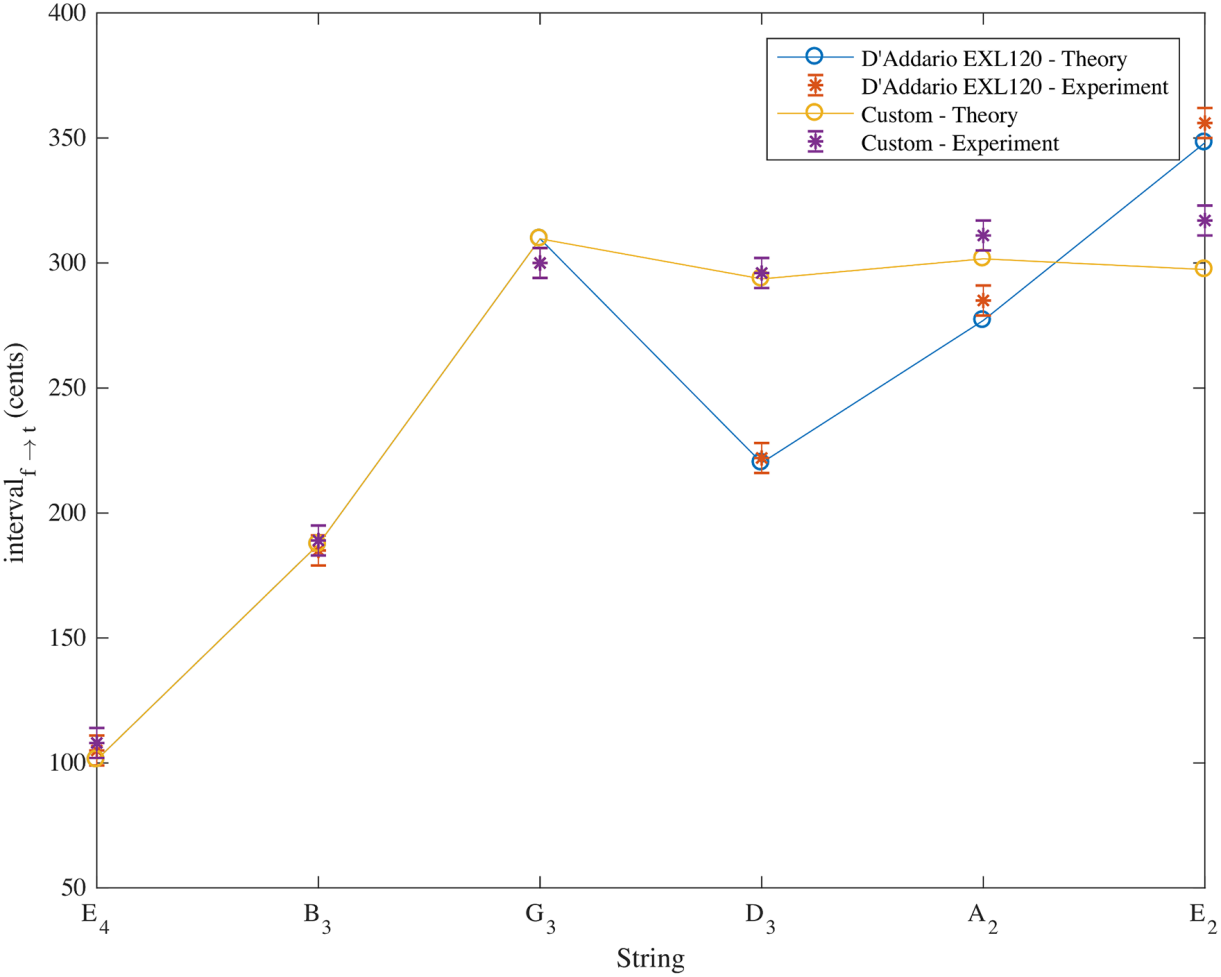
Theoretical and experimental pitch deviations in cents (with respect to the open string pitches) for a full tremolo arm pull up on Fender Stratocaster with D’Addario EXL120 Super Light 9-42 strings and with custom strings made by Newtone Strings to the author’s specification. Results are taken from Tables 2 and 6.

The strings were tested for their pitch deviations and then destructively tested to obtain the mass ratio *τ* using Eq (20) as before. Experimental measurement of the custom string set loaded onto the Fender Stratocaster is compared with theory in Table 6. Agreement between the theoretical and experimental pitch deviations is within 20 cents for a pull up of order 300 cents and demonstrates a musically useful level of matching between pitch changes obtained for tremolo arm use for the G_3_, D_3_, A_2_ and E_2_ strings. A comparison of the theoretical and experimental pitch deviations on full pull up for the custom string set and for the D’Addario EXL120 Super Light 9-42 strings are shown in Fig 4.

**Table 6:** Theoretically predicted versus experimentally measured pitch deviation for a tremolo arm pull up by *θ* = 0.085 radians for Fender Stratocaster with custom strings made by Newtone Strings to the author’s specification.

Theoretical							Experimental
Starting Pitch	Δ*l*/*l*_*f*_ (%)	Δ*l* (mm)	*δ* (mm)	*δ*_*c*_ (mm)	*τ*	interval_*f* → *t*_ (cents)	interval_*f* → *t*_ (cents)
*E*_4_	0.683	4.43	0.692	0.556	1	101	108
*B*_3_	0.386	2.50	0.737	0.610	1	187	189
*G*_3_	0.245	1.59	0.807	0.688	1	310	300
*D*_3_	0.271	1.76	0.810	0.713	1.97	294	296
*A*_2_	0.250	1.63	0.749	0.681	3.25	302	311
*E*_2_	0.249	1.62	0.710	0.668	5.75	297	317

Dimensions and variables are taken from Table 1. The values of *τ* were taken from destructive testing of the strings and utilising Eq (20). Other equations used for the theoretical calculations were Eqs (24), (30), (31), (28) and (29). Experimental data was measured on the guitar noting pitch intervals on the Logic Pro X tuner (with the display giving results that were consistent to plus or minus 6 cents). All values are to 3 significant figures.

It is interesting to note that the low E string shows the biggest discrepancy between the theory and experiment in two of the three measured string sets. In calculating the theoretical results it was assumed that the distance of string stretch was based on the point of contact between the string and saddle. In doing so we ignore any effects due to the finite thickness of the cores and windings of strings, the slightly rounded shape of the saddles and the slightly rounded profile of the string nearby and any slipping of the strings at the saddles. Adding the thickness of windings into the calculation of the string stretch (giving larger string stretches for the thicker wound strings) would improve the agreement between experiment and theory (giving 296, 306 and 306 cents for the D_3_, *A*_2_ and E_2_ strings respectively if the winding thickness given in the specifications is simply added in the distance to the pivot, *R*_*a*_).

## Temperature dependence

The vibration frequencies of metal strings on musical instruments are known to decrease with increasing temperature, necessitating retuning. In the case of steel (or steel cored) guitar strings the thermal coefficient of expansion of the steel in the strings [9] is significant, being in the range 11 × 10^−6^ m/mK <*a* < 13 × 10^−6^ m/mK. Here the units refer to meter of expansion per meter of initial length, per change in temperature in Kelvin (or, equivalently, change in Celsius). After a change in temperature of *dt* Celsius, the section of string formerly of length *l*_0_ when not under any tension would therefore have a rest length of *l*_0_ + *δ*_*a*_ when not under any tension, where

$$\begin{array}{c} \delta_{a}=al_{0}dt, \end{array}$$ (35)

with *a* being the thermal coefficient of expansion of steel. We will neglect the very small decrease in Young’s modulus with temperature.

The extension in the string (defined as the difference between its length under tension and its length if all tension was removed) therefore changes from Δ*l* to Δ*l* − *δ*_*a*_. Following a similar derivation to Eq (28), this results in a ratio of sounding frequencies of

$$\begin{array}{c} \frac{\nu_{a}}{\nu_{f}}\approx \sqrt{1-(\frac{\delta_{a}}{\Delta l})}, \end{array}$$ (36)

where *ν*_*a*_ and *ν*_*f*_ are the frequencies after and before the change in temperature respectively.

Using Eq (35), the interval in cents due to the temperature change by *dt* degrees Celsius thus becomes:

$$\begin{array}{c} {\text{interval}}_{f\to a}\approx 1200\log_{2}\left(\sqrt{1-(\frac{1-\frac{\Delta l}{l_{f}}}{\frac{\Delta l}{l_{f}}})a.dt}\right). \end{array}$$ (37)

**Fig 5:**
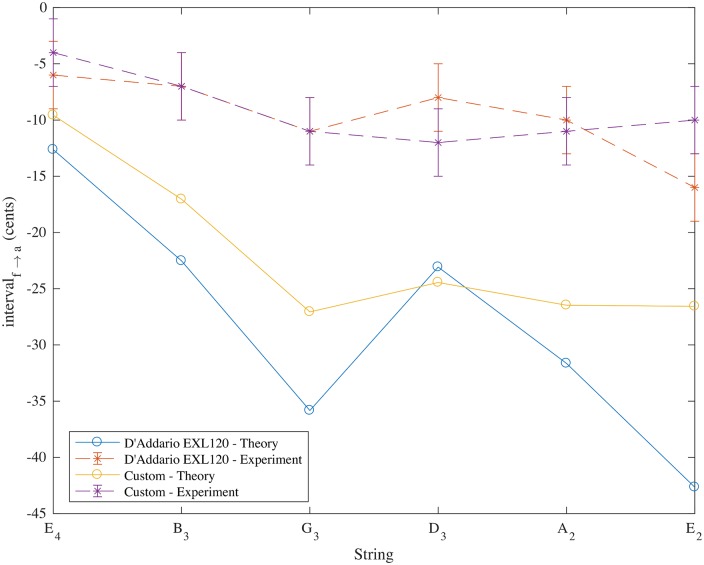
Theoretical and experimental pitch deviations in cents after an ambient temperature increase of 8.3°C for D’Addario EXL120 Super Light 9-42 strings and after an ambient temperature increase of 6.3°C for custom strings made by Newtone Strings to the author’s specification. The theoretical prediction is based on Eq (37) with values taken from Tables 2 and 6.

Theoretical and experimental results are displayed in Fig 5 with the experimental data obtained by leaving the guitar in a room with at a cool temperature (averaging 15°C for these experiments) for over an hour and tuning the instrument, and then bringing the instrument into a room with a warm temperature (averaging 23°C) and remeasuring the pitch 5-12 minutes later. The temperature at the location of the guitar for each measurement was read from a Tenma 72-7712 thermocouple thermometer. When the D’Addario strings were used the temperature change between the room was 8.3°C while for the test on the custom strings, the change in temperature was 6.3°C, hence the larger theoretical pitch change shown for the D’Addario strings. For these measurements, the tremolo screws were tightened so that the bridge plate was flush with the body in order to minimise the effect of movement at the bridge. Nonetheless, the results show that the experimentally measured pitch changes are only around a half to a third of those predicted by the simple theory which doesn’t account for the flexibility of the instrument body and its response to changes in string tension, temperature and humidity. It is, however, clear that strings with lower engineering strains are generally correctly predicted to have a higher sensitivity to temperature changes and thus the low E string (E_2_) on the standard D’Addario descends in pitch to the greatest extent and the low E (E_2_) in the custom set is improved in this regard, with the strings with close to equal engineering strain staying close to being in good relative tuning to one another with temperature changes.

## Conventional bending and feel

Conventional string bends involve pressing the string just behind a fret and sliding the finger parallel to the fret to support the string in a triangular shape with increased total length (and forcing other strings sideways to make room as necessary), producing an increase in tension and thus pitch [1]. Strings such as the custom set described above (designed according to the principle of close to equal engineering strains) will have a closer to equal sensitivity to pitch adjustments than strings of conventional specifications and this applies not just to tremolo arm movement, but also due to conventional string bending. In order to demonstrate this effect, the distance required to produce a whole tone pitch increase due to conventional string bending at the 12th fret was measured using the Logic Pro X tuner for assessing pitch change and digital callipers to measure distance changes. The custom D_3_ string described above (featuring 4 thousandths of an inch winding on a 16 thousandths of an inch core) required the string to be displaced along the fret by a distance of 12.5 ± 0.5 mm for a whole tone bend while the D_3_ string from the standard D’Addario EXL120 set required a larger displacement of 14.7 ± 0.5 mm. In comparison, the unwound G_3_ strings made by D’Addario and by Newtone Strings both had a core of 16 thousands of an inch and both required a displacement of 12.5 ± 0.5 mm. Since there is only around 9 mm between the strings when at rest, the small but measurable difference in displacement amounts to a noticeable improvement, matching the displacement required for conventional pitch bending when strings with approximately matched engineering strains are used.

## Conclusions

The theoretical pitch variation with tremolo arm usage for electric guitar strings has been derived and experiment matches theory with a mean error in cent values of less than 3% and a maximum error of 20 cents for changes of order 300 cents (or less than 7%), thus agreeing to within the experimental error inherent in precise measurement of the point of contact between the string and saddle with respect to the pivot. Setting out the engineering strains involved in tensioning wound and unwound strings was also an important aspect of this work, proving the assumption that the windings support negligible tension at pitch. Saddle height, the ratio of diameters/masses for core and winding and the distance of string behind the nut all have a measurable impact on relative tuning of strings under tremolo arm usage in agreement with the theory developed here.

In summary, unwound strings of higher sounding frequency will detune by a much smaller amount with a given control change assuming they are constructed of the same material (and, to the first approximation, this is independent of the string diameter assuming the tremolo claw is suitably adjusted to compensate for tension changes at pitch). The relative pitch interval through which wound strings detune due to tremolo arm movements or conventional string bends, on the other hand, is heavily dependent on the ratio of winding diameter to core diameter and this is a very effective control parameter in designing string sets that detune more evenly. It should be noted that while string sets are currently commercially available that have balanced tensions across all strings at rest, none of these are currently designed to have close to equal engineering strains or equal pitch deviations for control changes on standard hardware.

In a standard Fender Stratocaster style tremolo (whether a locking nut is fitted or otherwise), the 3rd, 4th, 5th and 6th strings may be given close to equal pitch deviations with tremolo arm use and conventional pitch bends through the use of similar core cross-sectional areas for those strings (assuming the diameter of the windings are chosen to give approximately equal tensions when tuned up to pitch). Temperature change related tuning problems, which introduce the most problematic tuning for the low E_2_ string in standard string sets, are also improved by using close to equal engineering strains. Other similar arrangements are possible, for instance giving matched sensitivities for wound strings and the B_3_ string for instance by approximately matching cores to the gauge of that string. While the paper focuses on the electric guitar, the analysis presented here could have applications to strings for other instruments including the bass guitar.

Ultimately, the choice of core and winding dimensions and materials are a matter of taste, with the relative tone quality, output level, tuning stability, durability and similarity to the strings used by famous performers all being of great importance to typical players and these topics are not the subject of the current paper. Nonetheless, the sensitivity of the strings to pitch bends (whether through tremolo arm use or standard pitch bends) is a crucial factor in the feel and musicality of the instrument and this paper sets out the conditions for understanding and potentially controlling this factor.

The University of St Andrews’ intellectual property rights contained in this publication are covered by a UK patent application.
